# Membrane-myofibril cross-talk in myofibrillogenesis and in muscular dystrophy pathogenesis: lessons from the zebrafish

**DOI:** 10.3389/fphys.2014.00014

**Published:** 2014-01-28

**Authors:** Maide Ö. Raeker, Jordan A. Shavit, James J. Dowling, Daniel E. Michele, Mark W. Russell

**Affiliations:** ^1^Division of Pediatric Cardiology, Department of Pediatrics and Communicable Diseases, University of MichiganAnn Arbor, MI, USA; ^2^Pediatric Hematology and Oncology, Department of Pediatrics and Communicable Diseases, University of MichiganAnn Arbor, MI, USA; ^3^Division of Pediatric Neurology, Department of Pediatrics, The Hospital for Sick ChildrenToronto, Ontario, CA, USA; ^4^Department of Molecular and Integrative Physiology, University of MichiganAnn Arbor, MI, USA

**Keywords:** myofibrils, T tubules, sarcoplasmic reticulum, sarcolemma, zebrafish, skeletal muscle

## Abstract

Striated muscle has a highly ordered structure in which specialized domains of the cell membrane involved in force transmission (costameres) and excitation-contraction coupling (T tubules) as well as the internal membranes of the sarcoplasmic reticulum are organized over specific regions of the sarcomere. Optimal muscle function is dependent on this high level of organization but how it established and maintained is not well understood. Due to its *ex utero* development and transparency, the zebrafish embryo is an excellent vertebrate model for the study of dynamic relationships both within and between cells during development. Transgenic models have allowed the delineation of cellular migration and complex morphogenic rearrangements during the differentiation of skeletal myocytes and the assembly and organization of new myofibrils. Molecular targeting of genes and transcripts has allowed the identification of key requirements for myofibril assembly and organization. With the recent advances in gene editing approaches, the zebrafish will become an increasingly important model for the study of human myopathies and muscular dystrophies. Its high fecundity and small size make it well suited to high-throughput screenings to identify novel pharmacologic and molecular therapies for the treatment of a broad range of neuromuscular conditions. In this review, we examine the lessons learned from the zebrafish model regarding the complex interactions between the sarcomere and the sarcolemma that pattern the developing myocyte and discuss the potential for zebrafish as a model system to examine the pathophysiology of, and identify new treatments for, human myopathies and muscular dystrophies.

## Myofibril organization

The highly ordered structure of vertebrate striated muscle is required for its normal function. Precise relationships between the contractile structures of the myofibril and the surrounding cytoskeletal and membrane compartments are maintained during contraction and relaxation. The cytoskeletal framework that surrounds and supports the myofibril links the myofibrils to each other and to specialized regions of the cell membrane and the sarcoplasmic reticulum involved in force transmission and excitation-contraction coupling, respectively (Hein et al., 2000). The importance of maintaining this structure is highlighted by the number of myopathies and muscular dystrophies that result when these relationships are altered (Kanagawa and Toda, 2006; Capetanaki et al., 2007; Kontrogianni-Konstantopoulos et al., 2009). Yet how this highly organized structure is established is not well understood.

What has been determined is that there is a close relationship between newly assembling myofibrils and the sarcolemma. Non-striated fibrils, representing immature or pre-myofibrils, were first noted immediately adjacent to the cell membrane in trout skeletal muscle [reviewed in Sanger et al. (2010)]. This finding has been confirmed in skeletal myotubes, in remodeling cardiac myocytes *in vitro*, in chick cardiac explants in primary culture, and in the developing human heart suggesting that interactions between the membrane and the myofibril promote the early steps of striated myofibril assembly in both heart and skeletal muscle (Terai et al., 1989; Kim et al., 1992; Rhee et al., 1994). The close association between the myofibril and the membrane [and through transmembrane adhesive contacts, the extracellular matrix (ECM)] may promote coordination of their maturation and organization during development and during adaptive remodeling. However, it has been difficult to model the development of the complex cellular organization of striated myocyte *in vitro* due to the importance of the three dimensional structure of surrounding cells and tissues in establishing the cell's internal architecture. *In vivo* studies have yielded important insights into cell and tissue organization but higher vertebrate models have been limited by the difficulty with monitoring the process in the developing embryo.

## The zebrafish as a model system for skeletal muscle development

The zebrafish (*Danio rerio*) is an excellent *in vivo* model system in which to examine the dynamic processes of new myofibril assembly and myocyte organization during development. As a vertebrate, the sarcomeric and cytoskeletal proteins involved in these processes are highly conserved with those in higher vertebrates including humans. Furthermore, the dynamics and progression of myofibril assembly has been demonstrated to be highly similar to that observed in mammalian models (Sanger et al., 2009). Zebrafish skeletal muscle forms from the myotome compartment of the developing zebrafish somite (Holley and Nusslein-Volhard, 2000). Prior to myoblast differentiation, a nascent boundary between the somites forms and ECM is deposited at regular intervals adjacent to the notochord (Raeker and Russell, 2011). Slow skeletal muscle myoblasts elongate, extending between the anterior and posterior somite boundaries. After elongation, the myocyte begins to differentiate, assembling new myofibrils and establishing a mature cellular structure. As the slow skeletal muscle cells migrate from their position adjacent to the notochord to the periphery of the somite, they stimulate differentiation of the fast skeletal muscle that will comprise the bulk of the myotome (Henry and Amacher, 2004). The somites first form at 10 hours post fertilization (hpf) and develop in an anterior to posterior direction at a rate of one every 20–30 min until 30–34 pairs have formed (Waterman, 1969). More mature somites are therefore located in the trunk as the new somites form in the elongating tail.

## Formation of the myotendinous junction

At the outset of myocyte differentiation and skeletal myofibrillogenesis in the zebrafish embryo, ECM deposition divides the developing mesoderm into distinct somites (Holley and Nusslein-Volhard, 2000). The ECM, which is enriched in fibronectin, is deposited at defined intervals along the notochord with extension of the matrix laterally and caudally to yield a “chevron-shaped” somite bounded by organized ECM (Raeker and Russell, 2011). The accumulation of ECM at the somite boundary requires a complex interplay of Eph-Ephrin signaling and α5-integrin-mediated matrix reorganization (Durbin et al., 1998). Eph-Ephrin signaling helps to confer myocyte polarity and specifies matrix deposition in defined domains. Integrins then mediate attachment of the cells to fibronectin within the somite boundary with the aid of focal adhesion kinase (FAK) (Crawford et al., 2003; Koshida et al., 2005; Julich et al., 2009). Loss of either α5-integrin or fibronectin destabilizes the somite boundary.

**Figure 1:**
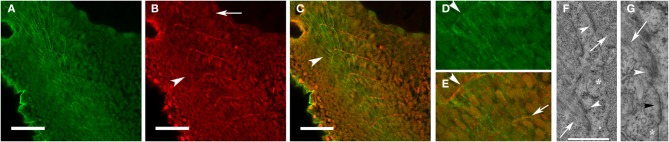
**Fibronectin organization at the somite boundary precedes the assembly and organization of new myofibrils**. Zebrafish embryos at 24 hpf were immunostained for fibronectin (red: **B,C,E**) and α-actinin (green: **A,C,D**). Fibronectin was organized at the somite boundary before the assembly of the first striated myofibrils (**B,C**: arrowheads) and becomes less abundant in the more mature boundaries (**B**: arrow). In the most recently formed somites, nascent myofibrils **(D,E)**, with a characteristic irregularly dotted pattern of α-actinin staining, extend between the recently formed fibronectin-containing nascent somite/myotome boundaries (**E**: arrow). **(F)** Insertion of actin filaments (arrows) into membrane-based adhesion domains at the somite boundary is associated with the accumulation of electron-dense material (arrowheads) on the inner and outer surfaces of the sarcolemma. **(G)** The membrane compartment at the somite boundary undergoes progressive development in preparation for myofibril assembly. Note the absence of electron dense material in an undeveloped region of the membrane (^*^) with deposition of some electron-dense material in a region without well-developed actin filaments (black arrowhead) followed by more accumulation of material in a region (white arrowhead) with definite insertion of well-developed actin filaments (arrow). Scale bars are 50 μm **(A–C)** and 2 μm **(F)**. **(D,E)** Are 3 × magnifications of corresponding regions in **(A,C)**. **(G)** Is a 2.5 × magnification of the image in **(F)**.

Elongating myoblasts attach to the matrix at the somite boundary and initiate new myofibril assembly at the site of attachment of the nascent myofibril to adhesion complexes within the sarcolemma [(Raeker and Russell, 2011) and Figure 1]. The assembly of new myofibrils is associated with progressive deposition of electron-dense material at the attachment site (Figures 1F,G) at the terminal ends of the myocyte, the zebrafish equivalent of the myotendinous junction (MTJ). As the somite boundary matures, fibronectin in the ECM is replaced by laminin and the cell-ECM contact sites become increasingly complex with the recruitment of talin, vinculin, and the dystrophin-dystroglycan complex (DGC) (Raeker and Russell, 2011).

The development and maintenance of the MTJ has been challenging to study in higher vertebrates. Studies in the zebrafish suggest that assembly and composition of the MTJ is very similar to the connections that form at the lateral sarcolemma called costameres and that disorders such as Duchenne muscular dystrophy, in which the costameric cell-ECM attachment complexes are compromised, also have defects of the MTJ that may be clinically relevant. Characterization of the *sapje* zebrafish, a model of Duchenne muscular dystrophy (Bassett et al., 2003), has demonstrated that dystrophin loss is associated with destabilization of the myocyte-MTJ attachment leading to detachment and cell death. Mouse models of impaired dystrophin-DGC function have likewise demonstrated structural abnormalities of the MTJ (Law and Tidball, 1993; Grady et al., 2003) and MRI studies in patients with DMD have demonstrated preferential myocyte loss and replacement fibrosis at the termination of muscle fibers at the MTJs (Nagao et al., 1991). Therefore, modeling of MTJ assembly and stability in the zebrafish may yield important insights into disease manifestations in patients with myopathies and muscular dystrophies, particularly those that involve the dystrophin-DGC.

### Relationship of the lateral sarcolemma to the underlying myofibrils

Costameres, the rib-like specialized membrane domains localized over the Z disks of the superficial myofibrils, were first described in the early 1980's (Craig and Pardo, 1983). Components of the costamere also overlying the M band but the structures are less consistent and less robust. It has been determined that the costameres are important sites for the lateral transmission of force generated by the underlying myofibrils and loss or damage of elements of the costamere, including the DGC, can lead to a range of muscular dystrophies [reviewed in Ervasti (2003)]. Yet, despite their importance, how these specialized membrane domains are developed and maintained with respect to the myofibril is not well understood. Much of what is known has been characterized in invertebrate (*D. melanogaster* and *C. elegans*) models [reviewed in Moerman and Williams (2006)]. Genetic screens for disordered sarcomere assembly and organization in *C. elegans* embryos led to the identification of mutants with a broad range of disorders affecting muscle structure and function. One class of mutants, the Pat (Paralyzed, Arrested elongation at Two-fold) embryonic-lethal developmental arrest mutants, were noted to have very early defects in myofibril assembly and were incapable of movement (hence the term, “paralyzed”). A second class of mutants, the Unc (Uncoordinated) mutants, is characterized by slow and/or irregular movements. Careful analysis of the mutants revealed a hierarchy of myofibrillogenesis that begins with the assembly of cell-matrix attachment complexes. Mutation or depletion of components of the transmembrane adhesion complexes, including α-integrin (Pat-2), β-integrin (Pat-3), integrin-linked kinase (Pat-4), and actopaxin (Pat-6), lead to a Pat or paralyzed phenotype in *C. elegans* and to comparable defects in *D. melanogaster* (MacKrell et al., 1988; Volk et al., 1990; Sparrow and Schock, 2009). The membrane-bound complexes organize the underlying ECM, including perlecan (Unc-52), and served as a scaffold for the assembly of the myofilaments, including the components of the sarcomere and the cellular cytoskeleton (such as kindlin/Unc-112, obscurin/Unc-89, and PINCH/Unc-97). Assembly and organization of new myofibrils progressed from the sub-sarcolemmal region to the cell interior.

**Figure 2:**
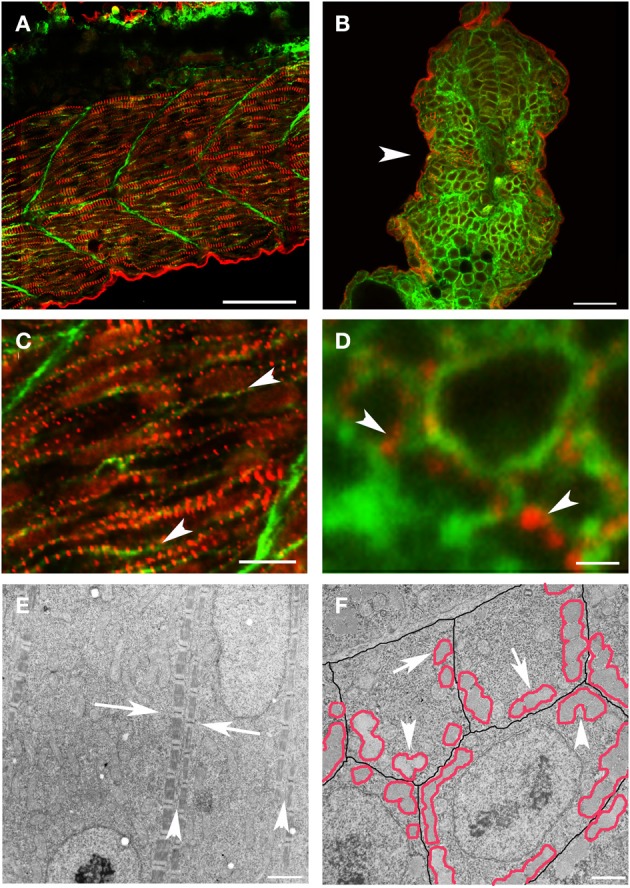
**New myofibril assembly occurs adjacent to the sarcolemma in skeletal muscle**. **(A–D)** Zebrafish embryos at 24 hpf were injected with lyn-GFP mRNA (which encodes for a GFP-fluorescent tagged protein that localizes to the cell membrane) and immunostained for α-actinin (red) and GFP (green) to mark the sarcomere and the sarcolemma respectively. Note that α-actinin dots (**C,D**: arrowheads) appear in a striated pattern immediately adjacent to the sarcolemma in both longitudinal optical **(A,C)** and transverse **(B,D)** sections. **(E)** Ultrastructural analysis reveals that there is a consistent relationship between new myofibrils that are forming on either side of the sarcolemma (**E**: arrowheads) and that there is a slight offset in the alignment of Z bands (**E**: arrows) across myocytes at this stage of development. In transverse sections **(F)**, note that the myofibrils (outlined in red) form in close proximity to the sarcolemma (outlined in black) with preferential clustering in regions where three cells intersect (arrowheads). There are examples of myofibrils (arrows) that are not paired with a myofibril in an adjoining cell, suggesting that the organization of myofibrils across myocytes may not be direct and therefore may require patterning of the extracellular matrix to transmit architectural information. Scale bars are 50 μm **(A)**, 20 μm **(B)**, 10 μm **(C)**, 1 μm **(D)**, 2 μm **(E)**, and 3 μm **(F)**.

However, it has been difficult to determine if a similar process occurs or is required in vertebrates. As previously noted (Kim and Ingham, 2009), new myofibril assembly in zebrafish skeletal muscle occurs in close apposition to the sarcolemma. In the zebrafish, new myofibrils are initiated at the terminal ends of the myocyte, in the sub-membrane compartment adjacent to the lateral sarcolemma of the elongated myocyte (Figure 2). Z disks of new myofibrils from adjacent myocytes were not in precise alignment but maintained a relatively consistent relationship such that the offset placed the Z disks from the myofibrils in one myocyte at approximately the A-I junction of the myofibrils in the adjacent cell [(Raeker and Russell, 2011) and Figure 2E]. The distance between the superficial myofibrils in adjacent myocytes was also maintained, suggesting that, very early in myofibril assembly, a cytoskeletal support network maintains relationships between the myofibril and specialized membrane domains that mediate attachment to the ECM (Figure 2E). The importance of the lateral membrane compartment and the submembrane cytoskeleton in scaffolding the formation of new myofibrils is supported by the preferential clustering of the cell's first myofibrils along the line of coaptation between three adjacent skeletal myocytes (Figure 2F).

### Reciprocal patterning of lateral membrane domains and newly assembled myofibrils

**Figure 3:**
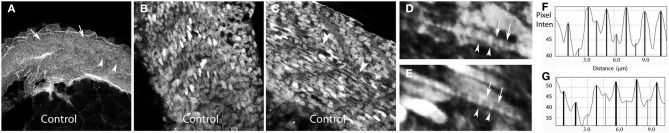
**β1-integrin redistribution occurs very early in myofibrillogenesis to pattern membrane domains overlying new myofibrils**. Zebrafish embryos at 20 **(A)** and 24 **(B–G)** hpf were immunolabeled for β1-integrin. Embryos demonstrated clustering of β1-integrin at new somite boundaries (arrows: **A**) with subsequent localization to membrane domains overlying new myofibrils in the more mature somites (arrowheads: **A**). **(B,C)** At the onset of myofibrillogenesis, β1-integrin localizes diffusely overlying the nascent myofibril before achieving a striated appearance with accumulation overlying the Z disk (arrowheads: **D,E**). Frequently a lower abundance and more diffuse accumulation of β1-integrin is noted at the mid A band (arrows: **D,E**). Line scans assessing pixel intensity notes a regular pattern of β1-integrin accumulation overlying the Z disks (black bars: **F,G**) with frequent smaller accumulations in the mid **(A)** band (grey bars: **F,G**). Note that, in general, the distance between Z disk accumulations of β1-integrin is less between Z disks without an intervening mid **(A)** band accumulation (**F**: far right; **G**: far left). Scale bars are 50 μm **(A)**, 20 μm **(B,C)** and 5 μm **(D,E)**.

To further characterize the relationship between the new myofibrils and the overlying cell membrane, we examined the organization of the sarcolemma into specialized domains during early myofibril assembly. Based on previous studies (Sparrow and Schock, 2009) and the order of organization of the MTJ, we postulated that integrins would have an early and primary role in organizing the membrane domains overlying the myofibril. Therefore, we performed immunolabeling of β1-integrin during zebrafish myofibrillogenesis. We noted that early during myoblast differentiation, β1-integrin localized to linear stripes along the long axis of the myocyte (Figures 3A–C). The staining was initially continuous but rapidly transitioned to a striated pattern that closely coincided with the organization of α-actinin into nascent Z disks (Figures 3D–G).

**Figure 4:**
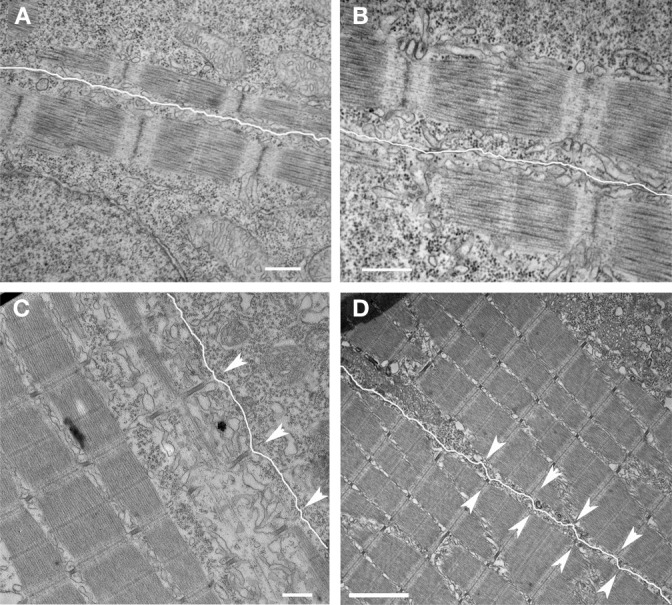
**Development of nascent costameres**. Skeletal muscle from zebrafish embryos at 20 hpf **(A,B)** demonstrate a consistent offset in the alignment of the Z disks across adjacent myocytes (sarcolemma highlighted in white). Note that the distance from the myofibril to the membrane is consistent over the length of the myofibril. By 72 hpf **(C,D)**, the membrane has a more undulated appearance and is closest to the myofibril overlying the Z disks (arrowheads) consistent with the maturation of costameric connections from the myofibril to the sarcolemma. Scale bars are 500 nm **(A–C)** and 2 μm **(D)**.

As with the MTJ, these immature cell-ECM contacts may recruit other components of the adhesion complex, including talin, vinculin, and dystrophin-DGC, to form a stronger and more permanent connection. It is likely that the early connection, as evidenced by the initially diffuse localization of β1-integrin, is fluid, allowing reciprocal interactions between the membrane contact and the underlying sarcomere to coordinate the patterning of both. As development proceeded, the sarcolemma became more closely associated with the myofibril at the level of the Z disk (Raeker and Russell, 2011). Combined with the slight offset of Z disks in adjoining myocytes, this gave the sarcolemma an undulated appearance (Figure 4). This ultrastructural change was noted prior to full maturation of the costamere as elements of the DGC, while evident at the somite boundary, had not yet localized to the lateral sarcolemma (Raeker and Russell, 2011). This suggests that fixation of the myofibril to the submembrane cytoskeleton, membrane-bound cytoskeletal and cell-matrix adhesion complexes occur prior to the maturation of the DGC and may scaffold its later assembly.

These findings suggest that specialization of the lateral sarcolemma overlying the superficial Z disks occurs very early in zebrafish myofibrillogenesis with specific localization of adhesion proteins and signaling molecules. The importance of the integrin-fibronectin attachments in anchoring the myofibril to the ECM was demonstrated in zebrafish embryos depleted of Tgfbi, an ECM protein expressed in response to TGF-beta signaling that binds to integrins (Kim and Ingham, 2009). Skeletal muscle depleted of Tgfbi formed normal appearing MTJs and myofibrils assembled in the subsarcolemmal space but the myofibrils did not remain closely attached to the sarcolemma and there was markedly reduced myofibril content. Similarly, zebrafish depleted of fibronectin demonstrated reduced myofibril content and organization (Snow et al., 2008). These studies suggest that integrin-mediated cell-ECM adhesion is an important stimulus for hypertrophic growth and an important regulator of myofibril organization but is not essential for myofibril assembly in zebrafish skeletal muscle *in vivo*.

The involvement of the ECM in the organization of myofibrils within the cell (Russell et al., 2000; Grosberg et al., 2011) may account for the coordination of the assembly process across adjacent cells resulting in the alignment of Z disks in register from one myocyte to the next. Alignment of sarcomeres across myocytes has been noted previously both in heart and skeletal muscle and is thought to have a role in optimizing force transfer and contractile response. Pathologic disease states such as end-stage heart failure (Schaper et al., 1991; Sharov et al., 1994), hypertrophic cardiomyopathy (Ferrans et al., 1972) and many myopathies (Milner et al., 1996; Marbini et al., 1998) and muscular dystrophies are all characterized by myofibril disarray and abnormal relationships between myocytes. The alignment process has not been well defined but has been attributed to patterning of the ECM by the myocyte (Caulfield and Borg, 1979; Robinson et al., 1987) or to direct patterning of adjacent cells by cell-cell contacts.

### Remodeling of myofibrillar clusters as adjacent myofibrils are bundled into larger units

**Figure 5:**
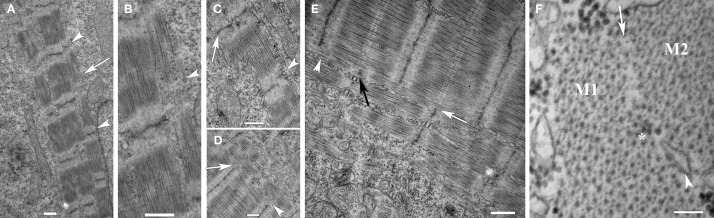
**Lateral integration of new myofibrils involves coordinate remodeling of the sarcomeres, the cellular cytoskeleton and the surrounding membranes. (A)** New skeletal myofibrils in a 20 hpf zebrafish embryo undergoing lateral fusion show progressive alignment at the level of the Z disks (arrowheads). Note the paucity of myofilaments in the intervening region between the Z disk involved in the active lateral incorporation (arrow) with intact sarcomeres on either end of the remodeling sarcomere. **(B)** A higher magnification of the image in **(A)** demonstrating the area of remodeling with interruption of the myosin filaments (arrowhead). **(C)** Newly assembled sarcomeres demonstrating recent alignment of the Z disks in register one end of the sarcomere (arrow) and misalignment at the other end (arrowhead). **(D)** Lateral integration of myofibrils with loss of defined sarcomere structure of the fusing myofibril (arrow) with progressive organization of the Z disk (arrowhead). **(E)** Higher magnification image of the image in **(D)** demonstrating remodeling of the T tubule/sarcoplasmic reticulum with loss of the membranes (white arrow) as the myofibrils fuse (black arrow). Note the disturbance of sarcomere architecture as the myofibrils fuse (arrowhead). **(F)** Transverse sections reveal that, as myofibrils (M1 and M2) fuse, the sarcoplasmic reticulum (arrowhead) withdraws from the line of apposition, the hexagonal array of myofilaments is disordered (^*^) on either side of the interface suggesting remodeling of the supporting cytoskeleton. Note that the new myofibrils are “separated” from the rest of the sarcoplasm by the membranes of the SR. Scale bars are 500 nm **(A–E)** and 100 nm **(F)**.

Within a given myocyte, as the peripheral myofibrillar clusters joined to form larger functional units, there was extensive remodeling of the sarcomere structure. If the adjacent myofibrils were slightly offset, the sarcomere structure remained intact, suggesting some capacity for myofibrils to “slide” into alignment during the bundling process. However, there were occasionally noted to be regions in which the offset between the Z disks of fusing myofibrils appeared to be too great to permit intact integration. In those regions, there were noted to be zones of transition in which existing myofibrils were dismantled and then reassembled. This was evident from the absence of intact sarcomeres in the transition zone between the part of the myofibril that had been incorporated into the adjoining myofibril and the part that was still unbundled (Figure 5). This was also noted on cross-sections where, in the areas of myofibrillar fusion, there was loss of the hexagonal arrangement of the actin and myosin filaments suggesting remodeling of the sarcomere's cytoskeletal support network in the transition zone.

One interesting feature of myofibril assembly and organization is how dynamic the process appears to be. Previous studies by the Sanger laboratory have revealed that sarcomeres are very dynamic structures in which there is a continuous protein turnover, even in intact, mature myofibrils (Sanger et al., 2005; Wang et al., 2005; Stout et al., 2008). Prior characterization of the myofibril organization and alignment process in primary cultures of chick skeletal myotubes in which Z disk alignment was described to progress by a “trial and error” that involved the disassembly of existing myofibrillar structures (McKenna et al., 1986). Proteosomal complexes strongly co-localize with new myofibrils (Foucrier et al., 1999, 2001) and mice lacking calpain-3, a cysteine protease, had significant abnormalities of sarcomere organization (Kramerova et al., 2004). Previous work from the invertebrate *C. elegans* model supports a role for ubiquitin-mediated proteolysis in sarcomere protein turnover with demonstration that (i) CSN-5, a component of the COP9 signalsome regulates levels of selected sarcomeric proteins (Miller et al., 2009) and (ii) MEL-26, an adapter that links cullin-3/ubuiquitin ligase complexes to the giant sarcomeric protein Unc-89, regulates thick filament organization (Wilson et al., 2012). The balance between chaperone-mediated protein assembly and ubiquitin-mediated protein degradation appears to have a central role in the assembly and remodeling of the myofibril in invertebrate models [reviewed in Willis et al. (2009); Kim et al. (2008)]. Our findings in the zebrafish support important role of protein complex assembly and degradation in myofibril organization and re-organization during development and suggest that the zebrafish may be an excellent model in which to study these dynamic processes.

### Organization of the membrane compartments surrounding the myofibril

Myocyte organization extends beyond the myofibril and sarcolemma to involve the internal membrane compartments involved in excitation-contraction coupling. Muscle contraction depends on the release and re-uptake of calcium stores from specialized membrane compartments to initiate contraction and permit relaxation respectively. Like the other elements of the myocyte, these membrane compartments, which include the junctional sarcoplasmic reticulum and invaginations of the sarcolemma called T tubules, are organized with respect to the underlying myofibril. As with other elements of the myocyte organization process, how these membrane compartments link to and become precisely situated over specific sarcomere domains is not well understood. Since these structures only exist in vertebrate muscle and the timing of their appearance during development varies between vertebrate species and between cardiac and skeletal myocytes, it has been very difficult to identify the structural elements required for their formation.

**Figure 6:**
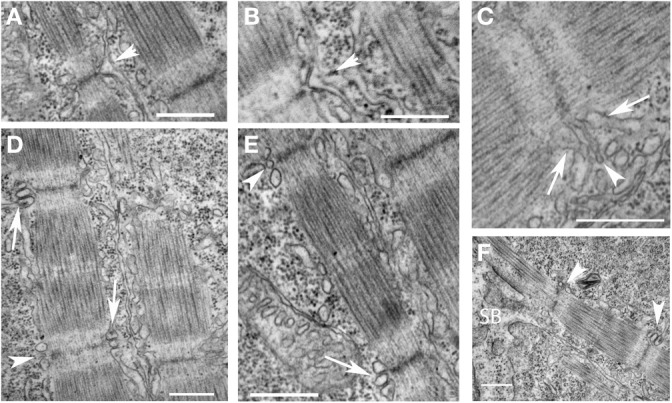
**Early organization of T tubules and junctional SR around newly-formed myofibrils. (A,B)** T tubules formed from invaginations (arrowheads) of the sarcolemma overlying the Z disks of newly formed myofibrils. **(C)** The T tubules (arrowhead) were often initially quite narrow initially before increasing in volume later in development. The sarcoplasmic reticulum (arrows) organized on either side of invaginating T tubules but did not initially appear to be in direct contact. **(D)** Triads (two junctional SR domains surrounding a T tubule) formed overlying the Z disk of newly formed myofibrils progressing from immature structures with small, round T tubules (arrowheads) to more mature structures with larger, oval T tubules in close apposition to the junctional SR (arrows). **(E)** A higher magnification image of a new myofibril with an immature (arrowhead) and more mature (arrow) triad at either end of the sarcomere. **(F)** Triads (arrowheads) develop around newly formed Z disks as new sarcomeres are added adjacent to the somite boundary (SB). Scale bars are 500 nm.

We used electron microscopic analysis to characterize the formation of the T tubules and sarcoplasmic reticulum during development. Unlike mammalian cardiac myocytes in which mature T tubules are frequently not noted until after birth, T tubules were noted, albeit not consistently, at the level of the Z disk in the newly formed myofibrils shortly after the appearance of mature sarcomeres (Figure 6F). T tubules appeared first as finger-like invaginations of the sarcolemma and later increased in cross-sectional area (Figures 6A–E). The sarcoplasmic reticulum was first diffusely localized in subsarcolemmal space and appeared more specialized on the cytoplasmic side of new myofibrils where junctional domains were noted associated with the T tubules. As was noted with the myofibrils, extensive remodeling of the T tubules and sarcoplasmic reticulum were noted in regions where adjacent myofibrils were bundling into larger units. Taken together with the findings from previous studies, our findings suggest that T tubular architecture is directly dependent on the cytoskeletal support network for the myofibril, and that the components of the cytoskeleton that are directly involved in anchoring the transverse domains of the T tubules are closely related to and may participate in the transverse alignment of myofibrils in register. Furthermore, the early formation of the T tubules in zebrafish skeletal muscle suggests that, even in species and tissues where T tubules form much later in development, the cytoskeletal network required to support T tubules may be present very early in new myofibril assembly. This network reorganizes to permit the lateral integration of myofibrils during new myofibril assembly and during adaptive cellular remodeling, leading to a loss of the transverse arrangement of the T tubules during these processes. Linkage of the T tubule system to the cytoskeleton may occur through spectrin (Messina and Lemanski, 1991), a sub-membrane cytoskeletal protein, that directly interacts with membrane-bound elements including ankyrin (Bennett and Stenbuck, 1979). Interaction between ankyrin and spectrin may help form a site of membrane anchorage to help nucleate the assembly of new myofibrils (Bennett et al., 2004) and contribute to the lattice that supports the T tubules (Messina and Lemanski, 1991). It has been postulated that ankyrin within the sarcolemma and the subsarcolemmal spectrin cytoskeleton are involved in establishing the membrane microdomains overlying the superficial myofibrils (Bennett et al., 2004).

### Modeling of human myopathies and muscular dystrophies

The ability to study dynamic relationships between adjacent myofibrils and between myofibrils and the surrounding membrane domains has encouraged the increased utilization of the zebrafish model for the understanding of the pathogenesis human myopathies and muscular dystrophies. Several muscular dystrophy mutant strains of zebrafish have been identified including those with mutations in dystrophin (*sapje*), laminin α2 (*candyfloss*), and dystroglycan (*pachytail* and *dag1*^*hu*3072^) (Bassett et al., 2003; Hall et al., 2007; Gupta et al., 2011) while several other genes causing muscular dystrophy have been disrupted by antisense morpholino approaches [reviewed in Gibbs et al. (2013a)]. One key phenotypic feature of many muscular dystrophy mutant zebrafish strains is disturbed birefringence of the skeletal muscle. The transparency of the zebrafish embryo enables direct visualization of skeletal muscle under polarized light. In wild-type embryos, the near crystalline structure of the ordered sarcomeres results in the unequal refraction of polarized light as it passes through skeletal muscle of the zebrafish trunk and tail, a property called birefringence. The intensity of the birefringence is reduced in the settings of myopathies and muscular dystrophies in which the myofibrillar architecture is disturbed. Therefore, measurements of the birefringence of the skeletal muscle of zebrafish can be used to assess the severity of myopathic changes in models of myopathy and muscular dystrophy, enabling the high-throughput assessment of disease severity and response to treatment.

In addition to myofibrillar structural integrity, muscular dystrophy mutant zebrafish also show marked changes in sarcolemmal integrity, an important hallmark of many muscular dystrophies. Loss of sarcolemma integrity has been demonstrated in DGC-deficient and in laminin beta2-deficient zebrafish models (Hall et al., 2007; Jacoby et al., 2009; Lin et al., 2011). The importance of sarcolemma integrity in muscle fiber maintenance is supported by the findings that morpholino disruption of one of the key proteins in the muscle membrane repair pathway, dysferlin, loss of which causes LGMD 2B and Myoshi myopathy in humans, also causes disrupted birefringence similar to DGC-deficient dystrophic zebrafish (Kawahara et al., 2011a). Zebrafish models should provide new insights into the relatively unknown plasma membrane repair pathways in muscle, because the optical transparency of zebrafish embryos renders the whole muscle amenable to high resolution real time microscope imaging to monitor the repair process (Roostalu and Strahle, 2012).

Zebrafish are becoming an increasingly important tool for screening for new therapeutic compounds. The validity and feasibility of performing high throughput screens for muscular dystrophy using zebrafish is supported by recent work of Kunkel and colleagues, who used birefringence to screen the Prestwick chemical library for compounds that restored muscle architecture in a zebrafish DMD model (Kawahara et al., 2011b). Future screens in zebrafish that utilize functional outputs such as swimming behaviors, as described by Kuwada and colleagues (Hirata et al., 2004; Horstick et al., 2013), may yield important new targets for therapy of muscular dystrophies that not only improve muscle structural integrity, but also improve muscle weakness and dysfunction seen in human patients that is independent of mechanical injury and loss of sarcolemma integrity (Gumerson et al., 2010, 2013).

### Application of genome editing techniques for manipulation of the zebrafish genome

Targeted germline modifications in the murine genome has enabled production of both “knock-out” and “knock-in” mice, allowing the development of animal models to study a wide range of human disease processes. However, gene targeting in the mouse can be expensive and time-consuming and the murine model may not be as amenable as the zebrafish to study dynamic processes that occur during development. Yet, until recently, the targeting of specific genes in zebrafish for the creation of disease models (i) was limited to morpholino antisense RNA-mediated inhibition of gene expression (which can cause non-specific toxic effects on development) or (ii) were the result of large, random mutagenesis screens where the strain harboring the mutation in the gene of interest needed to be identified by an exhaustive screening or specific phenotype manifestation. More recently, genome editing nucleases have been identified and used to help create specific disease models in the zebrafish. These nucleases have the capability to bind a specific DNA sequence and induce a double-stranded DNA break at that site. The inclusion of a homologous DNA fragment facilitates recombination and thus “edits” the genomic locus. In the absence of a repair template, non-homologous end-joining occurs, resulting in insertions or deletions, and subsequent frameshift mutations and/or truncations. In the presence of a repair template, the targeted DNA sequence can be replaced by one that contains a specific mutation, allowing the introduction of known or suspected disease-causing mutations into specific functional domains. There are two gene editing strategies, TALENs and CRISPR, that have emerged. TALENs (transcription activator-like effector nucleases) use DNA binding domains derived from a plant pathogen (Joung and Sander, 2013) and have been shown to be efficient at producing knockouts (Huang et al., 2011; Sander et al., 2011) and *in vivo* homologous recombination (Bedell et al., 2012; Zu et al., 2013) in zebrafish. CRISPR (clustered regularly interspaced short palindromic repeats), which works by a slightly different mechanism, is a set of genomic repeats found in bacteria and archaea that function as a system of adaptive immunity (Wiedenheft et al., 2012). As opposed to TALENs, CRISPR recognizes target sites using short guide RNAs that anneal to complementary genomic DNA sequences and recruit the Cas9 nuclease to effect cleavage (Blackburn et al., 2013). CRISPR has been found to be effective at generating knockouts (Hwang et al., 2013a), homologous recombination (Chang et al., 2013; Hwang et al., 2013b), and chromosomal deletions and inversions (Xiao et al., 2013). With these novel tools, rapid, efficient and inexpensive manipulation of the zebrafish genome can now be performed to create models of human disease.

### Use of the zebrafish for the identification of novel therapies

The high degree of molecular and structural identity, combined with the rapid development and large clutch size, makes zebrafish an ideal system for *in vivo* drug discovery to identify novel therapies. Drug discovery efforts can be largely divided into two approaches: targeted compound screening evolved from hypothesis-driven experimentation and large scale drug screening. Several previous studies have highlighted the utility of the zebrafish for targeted compound screening in models of human muscle diseases. Henry and colleagues demonstrated the ability of NAD+ to prevent dystrophic changes in DGC—or integrin α7—deficient zebrafish (Goody et al., 2012). Based on studies showing abnormal neuromuscular junctions in zebrafish with model of dynamin-2 centronuclear myopathy, acetylcholinesterase inhibitors were identified as modulators of the motor phenotype (Gibbs et al., 2013b). These examples reveal how the zebrafish model can serve as a quick and potent tool for testing specific chemical compounds. However, the real power of the fish lies in the ability to test drug libraries in order to discovery previously unidentified molecular and pharmacologic pathways.

Large scale drug screens have previously been not feasible *in vivo* in vertebrate models of human muscle disease. Now, however, with the characterization of numerous mutants in the zebrafish that mirror the genetics and the cellular abnormalities of their human disease counterparts, such screens have become a reality. This is particularly aided by the facts that zebrafish develop rapidly and *ex utero*, can be produced in large numbers, and readily absorb drugs presented in the media. Several screens have been successfully performed in models of a range of human diseases. The most high profile to date has been a screen by Zon and colleagues for melanoma, as they identified compounds using a zebrafish screen that are now in clinical trials for patients with melanoma (White et al., 2011). A single large scale screen has been completed and published for a zebrafish model of Duchenne Muscular Dystrophy (the sapje zebrafish) by Kunkel and colleagues (Kawahara et al., 2011b). The screen utilized birefringence, which is abnormal in all known zebrafish models of muscular dystrophy, as a read out. They identified 48 positive “hits” that prevented the abnormal birefringence in the sapje model, a list that included both known and newly identified drug targets for DMD. Subsequent analyses by this group based on their screen have focused on refining drug discovery related to cAMP-dependent protein kinase A modulation. Their results have laid the groundwork for future screens by (i) identifying known targets and thus validating the fish as a relevant model for screening drugs and (ii) discovering new target pathways and thus demonstrating the utility and power of zebrafish-based drug discovery studies.

## Summary

**Figure 7:**
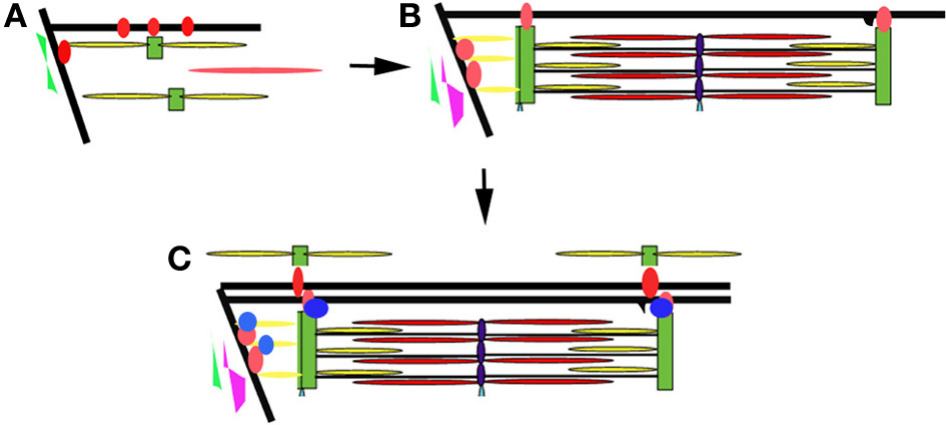
**Model of myofibril assembly and myocyte organization. (A)** The first myofibrils form at the insertion of actin filaments (yellow rods) and myosin filaments (red rod) at the terminal ends of the myocyte along the lateral sarcolemma. Integrins (red circles) are initially diffusely organized along the sarcolemma before concentrating at sites of actin insertion sites (terminal ends of the myofibril and overlying the Z disks of the superficial myofibrils) where they help organize the surrounding ECM including fibronectin (in green). **(B)** As myofibrillogenesis proceeds, integrins become more concentrated at contact sites and laminin (purple) is deposited along the somite border. Sarcolemmal invaginations that will eventually become the T tubular network form overlying the Z disks (black projection). **(C)** Additional adhesion complex proteins (blue) are recruited to stabilize and strengthen the contacts at the terminal ends of the myofibril. The patterning of the sarcolemma and the surrounding ECM results in the patterning of the sarcolemma and subsequently the newly assembling myofibrils of neighboring cells.

The developing zebrafish embryo has many properties that make it a very powerful model to study myofibril assembly and myocyte organization. Work in our laboratory and others suggest that there is a concurrent maturation and organization of the myofibrils and membrane compartments involved in lateral and longitudinal force transmission (the costameres and MTJs, respectively) and those involved in excitation-contraction coupling (the T tubules and sarcoplasmic reticulum) (Figure 7). The process appears to be dynamic and iterative, as assembled structures are disassembled and reorganized to align with other myofibrillar structures within the cell and within neighboring cells. Interactions between the sarcomere and the ECM help to determine tissue organization ensuring parallel transmission of contractile forces and resulting in optimal muscle performance. This work supports the characterization of sarcomeric, sarcolemmal, cytoskeletal and ECM remodeling as fully integrated processes during muscle development. As such, it will be important to characterize the response of each in the adaptive response to muscle injury or myopathy. Therapeutic targeting of the elements responsible for coordinating the relationships between these cellular compartments may promote preservation of muscle function or promote muscle repair in response to injury. The application of gene editing approaches and high throughput drug discovery and therapeutic testing will make the zebrafish an important resource in understanding the pathophysiology of a broad range of muscle disorders and in the identification of novel therapeutic strategies.

### Conflict of interest statement

The authors declare that the research was conducted in the absence of any commercial or financial relationships that could be construed as a potential conflict of interest.

## Abbreviations

SR: Sarcoplasmic reticulum
GFP: green fluorescent protein
ECM: extracellular matrix
TGF: transforming growth factor.
